# A Case of Suspected T-cell Intravascular Lymphoma Mimicking Multiple Hepatic Abscesses and Adult-Onset Still’s Disease

**DOI:** 10.7759/cureus.47534

**Published:** 2023-10-23

**Authors:** Yutaka Ohjino, Kasumi Nishikawa, Nozomi Nishikura, Chiaki Sano, Ryuichi Ohta

**Affiliations:** 1 Family Medicine, Yonemori Hospital, Kagoshima, JPN; 2 Family Medicine, Shimane University Hospital, Izumo, JPN; 3 Community Care, Unnan City Hospital, Unnan, JPN; 4 Community Medicine Management, Shimane University Faculty of Medicine, Izumo, JPN

**Keywords:** drug toxicity, atypical presentation, skin biopsy, nonspecific symptom, palliative care, differential diagnosis, diagnostic challenge, elderly patient, t-cell intravascular lymphoma, hyperferritinemia

## Abstract

Diagnosing hyperferritinemia can be challenging due to the nonspecific nature of symptoms and various potential causes. This case report discusses the intricacies faced when diagnosing an elderly individual with hyperferritinemia, which eventually led to a specific diagnosis. An elderly patient presented with two months of fatigue, fever, and malaise, initially receiving several diagnoses. Despite some treatments, the patient’s condition worsened, prompting further evaluation. Further investigation revealed a rare diagnosis. The progression of the disease led to the decision of palliative care. This case emphasizes the challenges in diagnosing elderly individuals and the importance of comprehensive follow-up and broad differential diagnosis. The need for a thorough investigation in the face of specific indicators is underscored. The patient’s non-response to certain treatments required the consideration of less common conditions in the differential diagnosis. The case offers insights into addressing treatment suitability and making necessary adjustments. The situation underscores the need for careful evaluation, especially in unusual presentations, and the importance of timely conversations about care options. Lessons from this case assist healthcare professionals in understanding and managing intricate presentations in the elderly.

## Introduction

Extreme hyperferritinemia is diagnosed with serum ferritin levels greater than 10,000 ng/mL and has a variety of causes [1]. Hemophagocytic syndrome (HPS) is one of the causes of extreme hyperferritinemia and requires special attention due to its high mortality rate and requisite for prompt treatment [2]. HPS results from the abnormal activation of macrophages and is characterized by the abnormal intake of blood cells [3]. A serum ferritin level of 10,000 ng/mL or higher has a high diagnostic value for HPS, with a reported sensitivity of 90% and specificity of 96% [3]. However, not all HPS/hemophagocytic lymphohistiocytosis (HLH) patients meet the diagnostic criteria, and approximately 7% of patients have ferritin levels of less than 500 ng/mL. Therefore, the diagnosis can be challenging. Nevertheless, early diagnosis and treatment of HPS are essential [2].

The differential diagnosis of extreme hyperferritinemia should be considered according to each patient’s clinical presentation as the frequency of its diagnosis varies with age. Diagnosing extreme hyperferritinemia is challenging because it rarely occurs in the elderly [4-6]. Herein, we report a case of hyperferritinemia in a 101-year-old patient with malaise and anorexia as the main complaints. The patient was initially diagnosed with multiple hepatic abscesses and adult-onset Still’s disease. However, treatment with steroids and tocilizumab was only partially effective. The patient’s condition gradually worsened, and serum ferritin levels gradually increased. A random skin biopsy revealed an abnormal extensive T-cell lymphocyte invasion of the skin, and the patient was diagnosed with T-cell intravascular lymphoma. This case report discusses the difficulties in diagnosing extreme hyperferritinemia and possible strategies to consider in older patients.

## Case presentation

A 101-year-old female presented to a rural community hospital complaining of fatigue and fever. Fatigue and mild fever had appeared two months before admission. One week before admission, she had visited the hospital with exacerbated symptoms. Laboratory tests revealed hyponatremia, likely resulting from a syndrome of inappropriate secretion of antidiuretic hormone triggered by viral infection. Therefore, she was administered acetaminophen (500 mg) tablets to treat her fever and oral salt intake for hyponatremia. The day before the hospital admission, she had been gardening for about 1.5 hours, felt ill, and had to rest. On the day of admission, she continued to have general malaise and mild fever and visited our hospital for further investigation. She had no history of butterfly-shaped erythema, sun sensitivity, skin hardening, periungual erythema, Raynaud’s phenomenon, purpura, skin rash, dry mouth, stomatitis, cough, or dyspnea. Her medical history included hypertension, dyslipidemia, diabetes mellitus, and chronic constipation. Four years before the hospital visit, prednisolone (10 mg) was commenced for polymyalgia rheumatica for bilateral shoulder pain and was subsequently tapered to 1 mg daily. Her medication included amlodipine (5 mg daily) and rosuvastatin (2.5 mg daily).

Vital signs at admission were as follows: temperature of 37.3°C, heart rate of 84 beats/minute, blood pressure of 126/66 mmHg, SpO2 of 95% (room air), and respiratory rate of 16 breaths/minute. Physical examination revealed clear consciousness, tenderness in both shoulder joints, and grasping pain in the bilateral thigh muscle, without tenderness in the bilateral temporal arteries. No specific abnormalities were observed in the head, lungs, heart, abdomen, or back. No redness or purpura were observed on the skin. Chest radiography revealed no infiltration or pleural effusion. The initial laboratory data revealed elevated inflammatory markers of C-reactive protein and hyperferritinemia with elevated soluble interleukin 2 receptors (Table 1).

**Table 1:** Initial laboratory data eGFR, estimated glomerular filtration rate; CK, creatine kinase; CRP, C-reactive protein; TSH, thyroid-stimulating hormone; Ig, immunoglobulin; SARS-CoV-2, severe acute respiratory syndrome coronavirus 2; C3, complement component 3; C4, complement component 4; MPO-ANCA, myeloperoxidase antineutrophil cytoplasmic antibody; CCP, cyclic citrullinated peptide

Parameters	Level	Reference
White blood cells (× 10^3^/μL)	7.9	3.5-9.1
Neutrophils (%)	78.9	44-72
Lymphocytes (%)	11.7	18-59
Monocytes (%)	8.3	0-12
Eosinophils (%)	0.5	0-10
Basophils (%)	0	0-3
Red blood cells (× 10^6^/μL)	3.18	3.76-5.50
Hemoglobin (g/dL)	12.3	11.3-15.2
Hematocrit (%)	34	33.4-44.9
Mean corpuscular volume (fL)	91.1	79-100
Platelets (× 10^4^/μL)	12.3	13-36.9
Total protein (g/dL)	5.4	6.5-8.3
Albumin (g/dL)	2.9	3.8-5.3
Total bilirubin (mg/dL)	0.3	0.2-1.2
Direct bilirubin (mg/dL)	1.7	0-0.4
Aspartate aminotransferase (IU/L)	139	8-38
Alanine aminotransferase (IU/L)	83	4-43
Alkaline phosphatase (U/L)	139	106-322
γ-Glutamyl transpeptidase (IU/L)	84	<48
Lactate dehydrogenase (U/L)	464	121-245
Blood urea nitrogen (mg/dL)	9.5	8-20
Creatinine (mg/dL)	0.81	0.40-1.10
eGFR (mL/minute/L)	48	>60
Serum Na (mEq/L)	125	135-150
Serum K (mEq/L)	4.2	3.5-5.3
Serum Cl (mEq/L)	92	98-110
Serum Ca (mg/dL)	8.2	8.8-10.2
CK (U/L)	35	56-244
CRP (mg/dL)	6.29	<0.30
Serum glucose (mg/dL)	142	70-110
TSH (μIU/mL)	1.14	0.35-4.94
Free T4 (ng/dL)	1.1	0.70-1.48
Serum ferritin (ng/mL)	22,481	12-249.9
HBs antigen	0	0
HBs antibody	0	0
HCV antibody	0.07	0
SARS-CoV-2 antigen	Negative	Negative
Antinuclear antibody		
C3 (mg/dL)	114	86-164
C4 (mg/dL)	28	17-45
MPO-ANCA (U/mL)	<1.	<3.5
Soluble interleukin 2 receptor (U/mL)	3,462	122-469
Urine test		
Leukocyte	Negative	Negative
Nitrite	Negative	Negative
Protein	Negative	Negative
Glucose	Negative	Negative
Urobilinogen	Normal	
Bilirubin	Negative	Negative
Ketone	Negative	Negative
Blood	Negative	Negative
pH	6.5	5-7.5

**Figure 1:**
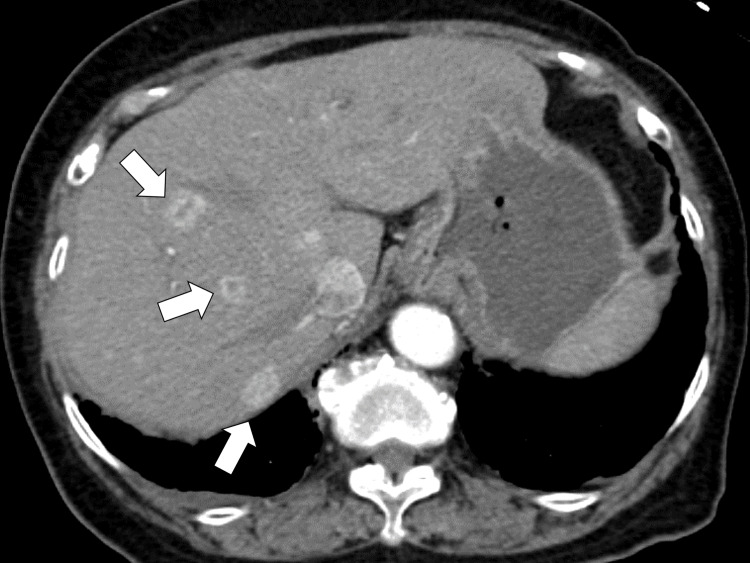
Enhanced chest-to-pelvic computed tomography showing multiple ring-enhanced lesions in the liver (white arrows)

To investigate the origin of the fever, enhanced chest-to-pelvic computed tomography was performed, and multiple ring-enhanced lesions in the liver were identified (Figure 1).

Blood and urine cultures revealed no bacterial infections. Consequently, she was diagnosed with multiple liver abscesses and treated with cefmetazole (CMZ) (4 g per day).

**Figure 2:**
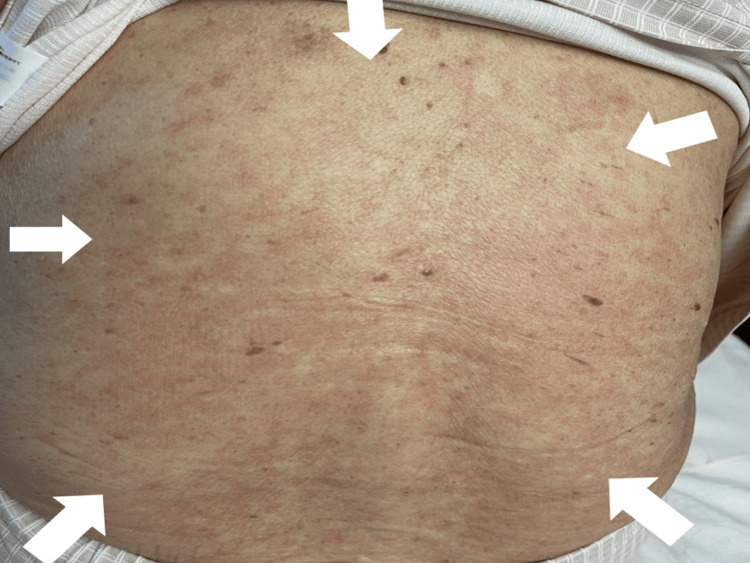
Erythema observed on the patient’s back (white arrows)

Her fever and fatigue gradually alleviated; however, on the eighth day of admission, her fever spiked to 39°C and only resolved the following morning. From the 10th day, the patient presented with facial oil, edema of the right eyelid, diffuse erythema of the trunk, and erythema on the anterior neck, back, and face (Figure 2).

Suspecting drug toxicity, CMZ was discontinued. Intravascular lymphoma and drug toxicity were suspected, and skin biopsies were performed, but they did not clarify any abnormalities. Generalized arthritis progressed, and anorexia appeared. A provisional diagnosis of adult-onset Still’s disease complicated by HLH was made, and prednisolone (20 mg) was initiated. Tocilizumab (300 mg biweekly) was administered to taper the prednisolone dose. After commencing prednisolone treatment, the serum ferritin level decreased to 7,341 ng/mL, systemic arthritis decreased, and by the 14th day, the prominent erythema of the back and anterior neck had disappeared.

**Figure 3:**
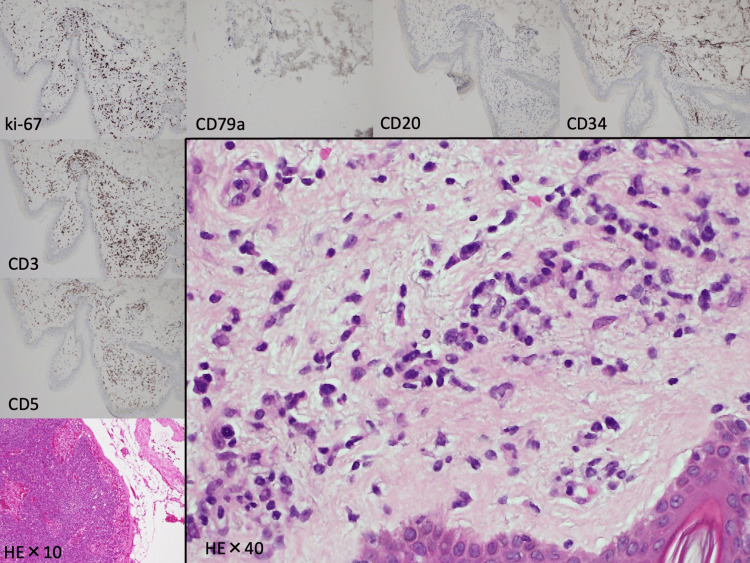
Random skin biopsy revealing an abnormal large T-cell lymphocyte invasion in the skin (H&E: ×10 and ×40): the predominating cells shown are negative for B-cell markers (CD20, CD34, and CD79a), with a particularly notable increase in CD3, CD5, and abnormal ki-67-positive lymphocytes H&E, hematoxylin and eosin; CD, cluster of differentiation

However, her consciousness deteriorated on the 15th day of admission, and her fever surged to 38°C. Sepsis was suspected, and tazobactam piperacillin was commenced at the dose of 13.5 g/day. On the 16th day of admission, an additional random skin biopsy revealed an abnormal extensive T-cell lymphocyte invasion in the skin. The predominating cells were negative for B-cell markers (cluster of differentiation (CD) 20, CD34, and CD79a), with a particularly notable increase in CD3, CD5, and abnormal ki-67-positive lymphocytes (Figure 3).

The patient was diagnosed with T-cell intravascular lymphoma. Through the discussion of further investigation, the families were hesitant to perform intensive procedures and treatments. Palliative care was performed after discussions with her family. Her symptoms worsened, and on the 22nd day, the patient died of disseminated intravascular coagulation.

## Discussion

This case report describes a 101-year-old female with prolonged fatigue and fever complaints. These symptoms were exacerbated over the week before admission. The patient was diagnosed with suspected intravascular T-cell lymphoma. This case is a valuable learning experience for clinicians in managing complex and evolving presentations, considering rare diseases, managing palliative care, and addressing the unique challenges faced when treating elderly patients.

This case highlights the complexities inherent in diagnosing elderly patients and the importance of meticulous symptom follow-up, given the nonspecific nature of their symptoms and the potential for multiple coexisting conditions. As this case report shows, the patient’s initial symptoms were vague, leading to an imprecise initial diagnosis. After an intensive investigation, a diagnosis of T-cell intravascular lymphoma was made. Considering this case and previous reports of intravascular lymphoma, clinicians should maintain a high index of suspicion and consider a broad differential diagnosis, particularly in elderly patients presenting with vague symptoms, such as fatigue and fever [7-9]. The evolving nature of the older patient’s symptoms and the emergence of new signs during hospitalization highlight the importance of close monitoring and re-evaluation, especially when the initial interventions do not lead to the expected improvements [10]. In addition, developing new symptoms following medications such as CMZ administration emphasizes the need to consider drug toxicity and re-evaluate treatment plans accordingly. In our case, fever and other symptoms persisted even after discontinuing the suspected medications, ruling out the possibility of drug side effects.

T-cell intravascular lymphoma is a rare disease, and its consideration in the differential diagnosis is crucial, especially in the presence of atypical presentations and when patients do not respond to conventional treatments. T-cell intravascular lymphoma is rarer than other lymphomas [9,11]. This case shows that T-cell intravascular lymphoma may not manifest systemic lymphadenopathy or abnormalities on peripheral blood smears. However, intravascular lymphoma can disseminate to various organs other than the lymph nodes. This can be difficult to diagnose, as the clinical presentations may mimic adult-onset Still’s disease and multiple liver abscesses [7]. In such cases, elevated inflammatory markers such as hyperferritinemia can serve as crucial clues to the underlying inflammatory or malignant conditions and should prompt further investigation. Hyperferritinemia can be caused by HPS and other hyperinflammatory conditions but lacks specificity for particular diseases [12]. For an effective diagnosis of intravascular lymphoma, meticulous investigation of the blood, bone marrow, skin, and other organs is required. However, as in this case, older patients and their families are hesitant to undergo further investigation; therefore, diagnosis is challenging. The diagnosis of intravascular lymphoma can facilitate advanced directives for patients and their families [9,13]. Family physicians approaching vague symptoms among older patients should suspect intravascular diseases and promptly investigate their patients while they remain independent or retain their activities of daily life [14].

This case highlights the importance of timely and sensitive discussions regarding palliative care options, particularly when dealing with the terminal diagnosis of an older patient. In our case, the patient was active and independent before admission. We, therefore, intensively administered the aforementioned medications. However, the need for intensive care, including intubation and central line medicated treatments, should be discussed with patients and their families to prevent undesirable intensive treatment and improve overall survival and quality of life [15,16]. A previous study showed that mutual understanding among medical staff, patients, and families can mitigate family bereavement after a patient’s death [17]. Therefore, early engagement with a patient’s family and a multidisciplinary approach to end-of-life care is essential.

## Conclusions

This study illuminates the complexities and challenges of diagnosing hyperferritinemia in elderly patients, as evidenced in the case of a 101-year-old female patient. The nonspecific nature of the patient’s symptoms and the potential for rare diagnoses (such as T-cell intravascular lymphoma) were shown, emphasizing the necessity for a meticulous follow-up and broad differential diagnosis. This case highlights the importance of considering drug toxicity and re-evaluating treatment plans. Moreover, the significance of hyperferritinemia as a diagnostic indicator and the importance of sensitive discussions pertaining to palliative care options were emphasized. Ultimately, this case report provides valuable insights for clinicians regarding the management of complex presentations in older patients.
